# Why are animal source foods rarely consumed by 6-23 months old children in rural communities of Northern Ethiopia? A qualitative study

**DOI:** 10.1371/journal.pone.0225707

**Published:** 2020-01-08

**Authors:** Mekonnen Haileselassie, Getachew Redae, Gebretsadik Berhe, Carol J. Henry, Michael T. Nickerson, Bob Tyler, Afework Mulugeta

**Affiliations:** 1 School of Public Health, College of Health Sciences, Mekelle University, Mekelle, Ethiopia; 2 Tigray National Regional State, Bureau of Science and Technology, Mekelle, Tigray, Ethiopia; 3 College of Pharmacy and Nutrition, University of Saskatchewan, Saskatoon, Canada; 4 Department of Food and Bioproduct Sciences, University of Saskatchewan, Saskatoon, Canada; McMaster University, CANADA

## Abstract

**Background:**

Animal source foods provide high-quality protein and essential micronutrients within the human diet and are of particular significance for the health and development of children. Despite the availability of domestic livestock in rural households of Ethiopia, the diets of children are often monotonous and mainly cereal-based with low energy and nutrient density.

**Objective:**

Explore barriers and facilitators for the consumption of animal source foods among 6–23 months old children from the rural communities of Northern Ethiopia.

**Methods:**

A community-based exploratory qualitative study design was conducted in July through September 2018. A total of eight focus group discussions (56 individuals) and twenty-four qualitative interviews were conducted with mothers who are lactating, fathers, health extension workers, nutrition, and agriculture experts. Purposive sampling technique was used to include study participants based on their potential relevance in delivering a wealth of information. Thematic analysis strategies, a method for identifying, analyzing, and reporting themes within data, were used to code and grouped into related families and synthesize the qualitative data.

**Results:**

Consumption of animal source foods among 6–23 months old children was very low and the home-reared livestock and their products were mainly used for market purposes. Animal products are consumed during special societal occasions since they are considered as luxury food rather than an essential part of daily children’s diet. Lack of nutrition knowledge, high cost of animal source foods, mothers’ workload to herd livestock, low household income, low milk production, the poor linkage between health and agriculture sectors, and social norms and beliefs were identified as common barriers. While the presence of nutrition experts, cooking demonstrations, in-kind credit programs, livestock ownership, and government-led stunting reduction programs were the facilitators for the consumption of animal source foods in the study communities.

**Conclusions:**

Reduced consumption of animal source foods inadvertently impacted dietary diversity of 6–23 months old children from the study communities. Thus, strengthening social and behavior change communication to promote the consumption of animal source foods, creating opportunities for women to own small livestock for household consumption and provide nutrition education on dietary restriction of animal source foods during religious periods among 6–23 months old children in the rural communities of Northern Ethiopia are recommended.

## Introduction

Animal based foods are the major source of quality protein and essential micronutrients of particular significance for the health and development of infants and young children [1–3]. Adequate intake of animal source foods (ASF) is found to be strongly associated with improved growth, cognitive function, physical activity levels, school performance, pregnancy outcome, and morbidity in young children [4–7]. Thus, the use of ASF can promote diet diversity and nutrition in young children [8, 9].

Ethiopia is known to have one of the largest livestock populations in the world [10]. However, the average per capita annual consumption of meat, egg, and dairy products is 4.6 kg, 0.2 kg and 16.7 kg, respectively which are lower than one-tenth of U.S. consumption [11]. As a result, complementary foods of children in the country often depend on monotonous diets, mainly based on staple foods such as cereals or tubers, which are characterized by low energy and nutrient density [12, 13]. In rural areas, ASF is typically consumed during special family/societal occasions as it is considered a luxury food rather than an essential part of the daily household diet [14, 15].

Child under-nutrition continues to be a major public health problem in Ethiopia [16, 17]. According to the preliminary findings of the Ethiopian Mini EDHS 2019, the prevalence of stunting, underweight and wasting in children under-five were found to be 36.8%, 21.1%, and 7.2%, respectively [18]. Similarly, the prevalence rate of anemia in children under-five was 53.8%. Zinc and vitamin A deficiencies were also public health problems in children and mothers in Ethiopia [16].

Considering the high prevalence of under-nutrition in children, improving access to the diversified diet through the consumption of ASF among infants and young children is critical. Currently, Ethiopia requires to speed up its efforts to attain the second National Nutrition Program (NNP II) target of reducing the status of stunting in under-five children from 40% to 26% by 2020 [19], and the Seqota declaration aims at realizing an end to childhood stunting by 2030 [20]. Thus, identifying the barriers and available resources for optimal consumption of ASF among infants and young children in the rural communities could support policy makers and implementers to craft effective interventions, strategies and hence achieving its targets. Thus, the purpose of this qualitative study was to explore the barriers and facilitators of consumption of ASF among 6–23 months old children in rural communities of Northern Ethiopia.

## Methods and materials

### Study setting

**Fig 1:**
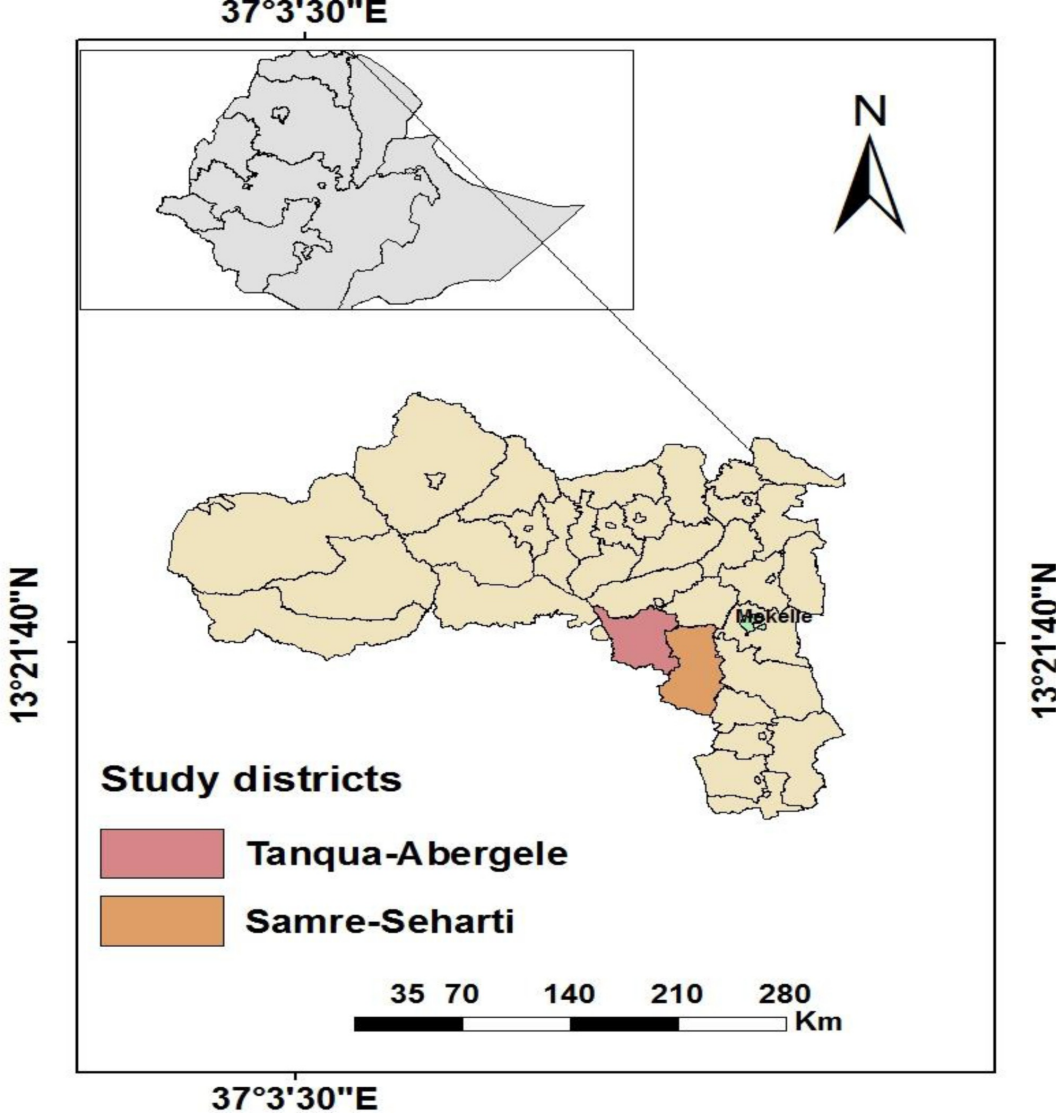
Study districts from the Tigray Administrative Region, Northern Ethiopia.

The study was conducted in two food-insecure districts of Tigray region namely Samre-Seharti, and Tanqua-Abergele which both are located along the Tekeze basin “Fig 1”. The total population of Samre-Seharti is 144,527 and 120,180 for Tanqua-Abergele. The districts are project areas for the Seqota Declaration interventions with a target of achieving an end to childhood stunting by 2030 [20], which calls for scaling up and intensification of the high impact nutrition interventions.

The study areas are characterized by smallholder mixed crop-livestock agriculture. Crop production consists mainly of tef (*Eragrostis tef*), wheat (*Triticum aestivum*), barley (*Hordeum vulgar*), chickpeas (*Cicer arietinum*) and peas (*Pisum sativum)*. The most common sources of ASFs raised by farmers include cattle, sheep, goats, and chickens. Livestock products (e.g. milk, eggs) and other economic services from the livestock (e.g. oxen draft power), goats, sheep, and chickens are often seen as sources of cash in times of financial constraints. The communities are largely Orthodox Christians where the consumption of ASFs during fasting occasions is strictly prohibited and the contamination of feeding utensils with the ASFs is a big issue.

### Study design

A community-based exploratory qualitative study was carried out to explore the barriers and facilitators of ASFs consumption among 6–23 months old children in the study area. This approach was chosen as it allows exploring the research topic with varying levels of depth [21]. Interview and discussion guides were used to facilitate the dialogue among selected groups and individuals from the study communities. Data were gathered from the study settings from July 2018 till September 2018.

### Sample size and sampling technique

**Fig 2:**
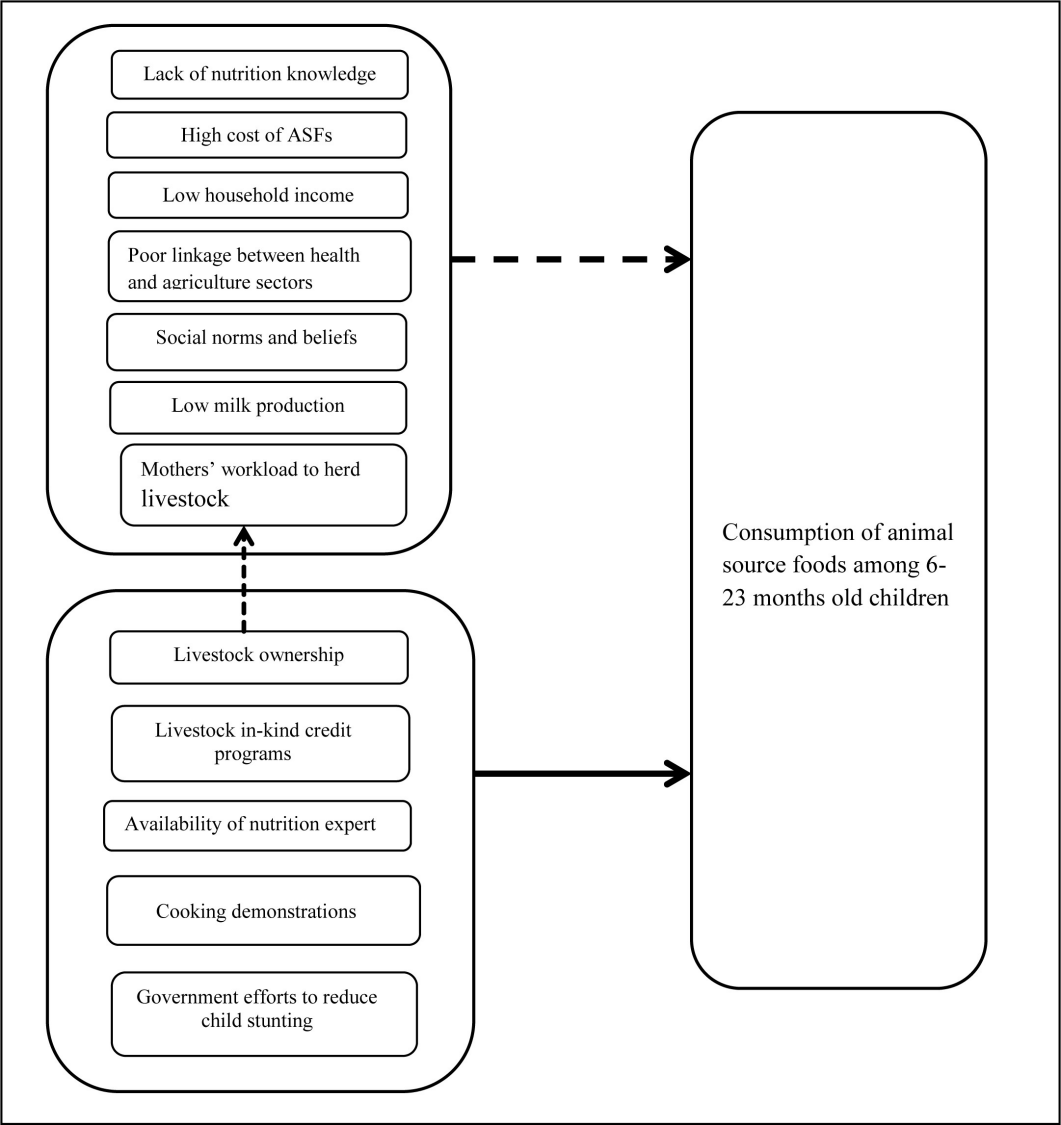
Schematic representation of the sampling procedure for the qualitative study in Tigray, Northern Ethiopia, 2019.

Districts namely Samre-Seharti, and Tanqua-Abergele were purposively selected as both districts were the potential candidate areas for Seqota Declaration in the Tigray region. Three Tabias (lowest administrative unit consisting of about 5,000 people) [22] were selected from each district randomly. The study Tabias were Tekleweini, Simret, and Jijke from Tanqua-Abergele district and Mariamoko, Adishishay, and Adikaela from Samre-Saharti district. Participants for focus group discussions (FGDs), key informant interviews (KIIs) and in-depth interviews (IDIs) were purposively selected as its main purpose was to select participants with adequate information on the issues to be discussed during the discussion and interview sessions [23]. Participants in FGD and IDI interview included parents (fathers and mothers) who had 6–23 months age children and at least one type of livestock in the household. Based on maximum variation purposive sampling technique, a range of participants were identified to represent the array of experiences and characteristics. Overall, a total of eight FGDs and twelve IDIs (fathers and mothers) and twelve KIIs (health extension workers, agriculture experts, and nutrition focal persons at various levels of the government structure) were included in the study “Fig 2”. The total sample size was determined on the basis of theoretical saturation (the point in data collection when new data no longer bring additional insights to the research questions) [23].

### Data collection methods

Qualitative data were collected from each FGD, KII and IDI participants. It was collected and analyzed by the principal Investigator (M.H.) who is a PhD student in public health and A.M. a nutritionist who has authored a number of qualitative studies. Study participants were selected depending on the potential relevance of the participants in delivering a wealth of information [24] about the barriers and facilitators to consume ASFs by infants and young children. Both FGDs and IDIs were held in the village’s health post and community development center where privacy and low background barriers for recording were conducive. Identification of interview and FGD participants were aided by local health and agricultural extension workers and Tabia leaders.

In-depth interview and discussion guides that cover the consumption barriers and facilitators of ASFs among infants and young children were used to collect the qualitative data. The guiding questions were prepared in English “S1 Table”, and then translated into the local language (Tigrigna) “S2 Table”. The guides were pre-tested in communities of a similar setting with the study communities and were revised accordingly. Daily debriefing sessions were undertaken during the data collection period to include new issues in the upcoming interviews. Face to face interview was carried out for the IDIs. The discussions were held in a circular seating arrangement where the participants could see face to face [25]. Each FGD consisted of 6–10 participants. The FGDs were facilitated by a moderator and note taker and took 50–80 minutes each. KIIs and IDIs were undertaken for a minimum of 30 minutes. Data were recorded via tape recorder, translated into English language and transferred to an encrypted personal computer for safety and privacy. Investigators reviewed the audio data from FGDs and IDIs and then directly translated from the vernacular language, Tigrigna, into English.

### Data analysis

The audio-recorded materials were transcribed verbatim in the local language Tigrigna and then translated into English by the investigator. Atlas ti. (version 7.5.4, Atlas. ti Scientific Software Development Mnbh, Berlin) qualitative data analysis software was used to store, manage and code all transcribed data. Transcripts were analyzed using both inductive and deductive coding [26]. Two investigators independently coded the data, and they sat together to assess the inter-coder reliability. In the case of dissimilarity, discussions were held until both reach into an agreement. Both investigators reviewed all the codes for consistency and construction of the teams. Consequently, similar codes were categorized based on the specific objectives and data collected. Thematic analysis approach was applied to analyze [27], and at times synthesize, the main topics of categorized data collected through interviews and discussions. Due attention was given to variation and similarities of views. Description of themes was supported by elaborative quotes. All texts under quotation represent direct voice of the study participants directly translated to the English language.

### Ethical consideration

The study was approved by Mekelle University‘s ethical review committee (Reference number: ERC 1432/2018) and permission to carry out the study was obtained from the Tigray Regional State Health Bureau. This qualitative research was conducted under standard ethical research procedures and took maximum care to protect the privacy and welfare of the study participants. All processes were made to uphold the confidentiality and privacy of study participants. Only the researchers had access to the study data, or to transcripts or recordings of the interviews and discussions. Informed consent was obtained from each study participant after they had been informed on the purpose of the study, benefit and risk, anonymity, confidentiality and their right to decline to answer any question or withdraw from the discussion or interview at any time. They were also requested to express their opinions and thoughts freely. Due to the literacy level of the FGD and IDI participants, verbal consent was preferred in our context without violating the ethical principles and it was approved by the ethical review committee.

## Findings

### Socio-demographic and economic characteristics of study participants

A total of 12 health extension workers/agriculture and nutrition experts, 19 fathers and 49 mothers who are lactating included in the in-depth interviews and focus group discussions. The age of the participants ranged between 22 and 53 years. All the participants were Orthodox Christian followers. All of the respondents mentioned crops and livestock as their major source of income. Goats were the most common livestock in the study communities. The detailed characteristics of the KII, IDI and FGD participants are shown in “Table 1”.

**Table 1:** Socio-demographic and economic characteristics of the qualitative study participants in the rural communities of Tigray, Northern Ethiopia, 2018.

Characteristics		IDIs (N = 12)		FGDs (N = 56)		KIIs (N = 12)*		
		Mothers (n = 6)	Fathers (n = 6)	Mothers (n = 43)	Fathers (n = 13)	HEWs	NFPs	AEs
Sex	Male	-	6	-	13	-	2	2
	Female	6	-	43	-	6	1	1
Average age of participants, years		31.2	35.8	29.1	38.2	26.5	31.7	42
Average family size of households		4.6	4.9	4.7	5.2	-	-	-
Average work experience, years		-	-	-	-	5.7	6.7	9.3
Educational level	No formal education	4	1	25	4	-	-	-
	Elementary and secondary	2	5	18	9	-	-	-
	College diploma	-	-	-	-	6	-	-
	Degree and above	-	-	-	-	-	3	3
Livestock ownership	Cattle	3.5	3.8	2.3	3.6	-	-	-
	Sheep	11.6	9.4	6.5	5.8	-	-	-
	Goats	12.8	12.2	7.8	6.2	-	-	-
	Chickens	5.2	3.8	4.9	6.7	-	-	-

*: HEWs–health extension workers; NFPs–nutrition focal persons; AEs- Agriculture experts.

### Perceived barriers and facilitators of ASFs consumption

**Fig 3:**
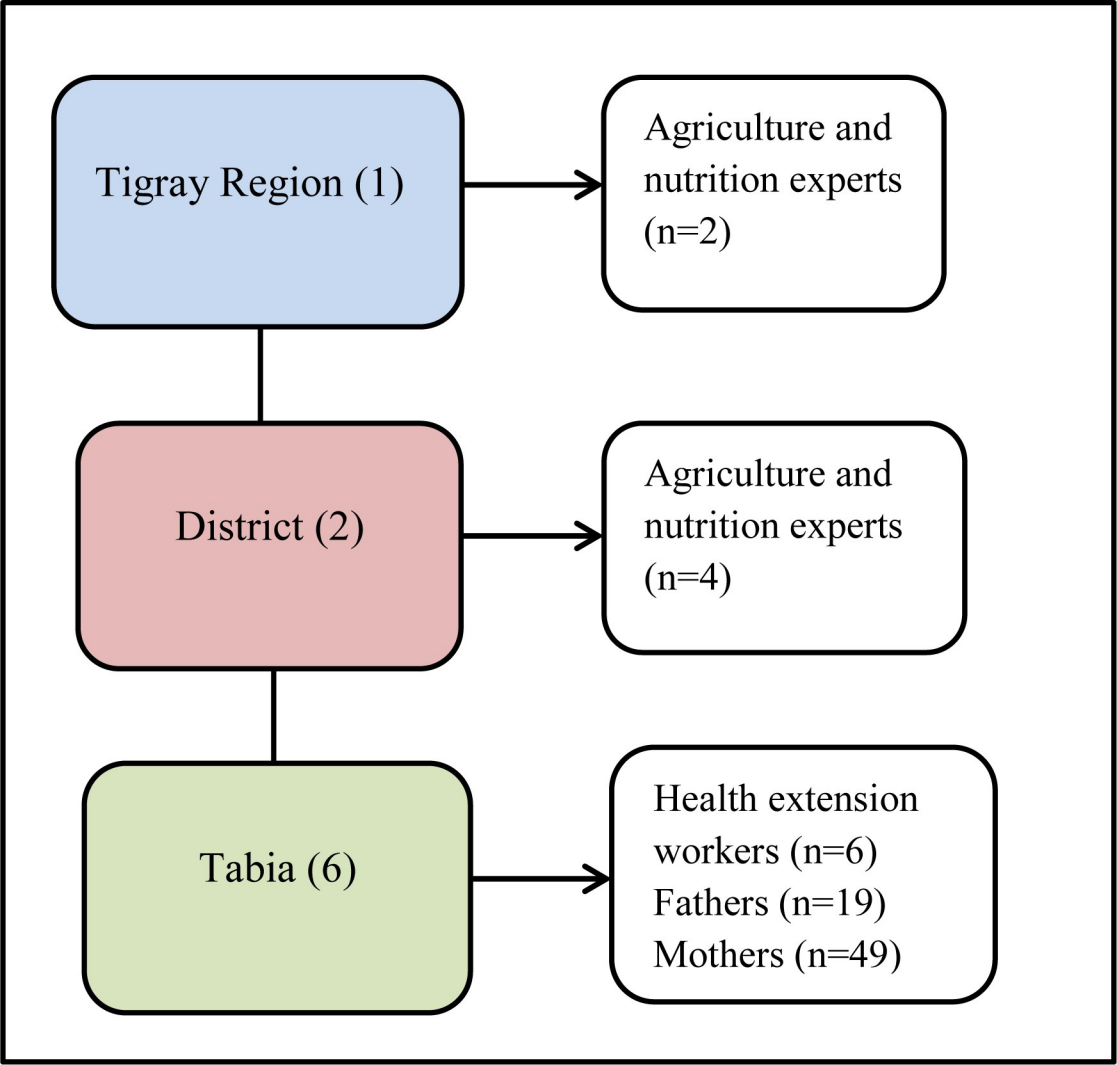
Conceptual framework describing the barriers and facilitators to the consumption of animal source foods among 6–23 months old children in rural communities of Northern Ethiopia. Note: the solid line indicates positive influence and dashed lines show the negative influence on the consumption of ASFs among 6–23 months old children. Livestock ownership has positive effects for the consumption of ASFs among children and it has also negative impact on mothers’ workload to herd livestock.

The findings revealed that livestock products as sources of cash/income, nutrition knowledge, cost of ASFs, mothers’ workload to herd livestock, household income, low milk production, the poor linkage between the health and agriculture sectors and social norms and beliefs were the barriers for the consumption of ASFs. Similarly, availabilities of nutrition experts, cooking demonstrations, government efforts to reduce child stunting program, in-kind credit program, and livestock ownership were identified to be the perceived facilitators of the consumption of ASFs in the study communities as shown in “Fig 3".

### Perceived barriers of the consumption of ASFs

#### Livestock products as sources of cash/income

Although most of the rural communities were producing livestock at home, they rarely provide ASFs to their children. Animal products were mainly used as sources of cash or income to the households as noted in the following transcripts.

*Any animal and animal product produced in our community is planned for the market purpose; for example, milk is not sold but it is not given for child in whole milk rather it is given in the form of skim milk after extracting the butter for the market purpose (FGD, mother, age 32 years)*.

The study participants mainly discussed about livestock in profit-making language through selling of the animal and animal products rather than food consumption in the community. Thus, the study communities produce livestock for market purpose and any ASFs are consumed mainly during holidays as stated in the following quotes:

*ASFs mainly meat are consumed during the holidays or on special occasion/festivities. For example, my family could get meat during the holidays of Ethiopian New Year, Christmas and Easter and when there are ceremonies such as a wedding and child baptism (IDI, mother, age 34 years)*.

Another FGD discussant said:

*Our diets are* cereal-based. *Injera with shiro (local stew from chickpea or pea powder) is our children’s common food*. *Animal source foods such as meat*, *eggs*, *or chicken are special holiday meals for our children (FGD*, *mother*, *age 38 years)*.

#### Nutritional knowledge

Though there is strong evidence for the role of increased consumption of ASF in promoting child health and nutrition, there are knowledge gaps on the importance of ASFs in the study communities. Participants expressed that one prominent concern is less awareness about the nutritional benefit of ASFs to children. An FGD had to say:

*We want to consume ASFs as it is delicious. However, we don’t know its nutritional benefit. I don’t know the importance of meat or egg for my children. What worries me most is the satisfaction of hunger. If the children fill their stomach either with egg or injera, it is ok with me as I consider both equally important (FGD, mother, age 38 years)*.

The participants also reported that there is an improvement in terms of the accessibility of food for their children as compared to previous times although no difference was observed in their strength and the health status of the parents and the children.

*…you are asking us to know whether our children consume egg or meat. But what is special now? We ourselves grew up without any access to meat, but still, we are strong. Why should we care more for meat if they get bread and injera? The only worry we have is our children should not get hungry regardless of the food type (FGD, mother, age 38 years)*.

On the other hand, few respondents reported that some literate young mothers were slowly changing their traditions of serving ASFs to their children, as explained in the following quote:

*ASF consumption among infants and young children is improving from time to time. My daughter who is 24 years old, and she has a two years old child and she gives him milk of goats and egg every day (IDI, male, age 56 years)*.

#### Cost of ASFs

In addition to the knowledge gaps on the nutritional importance of ASFs, the price of animals and ASFs were so costly that their consumption is perceived as a luxury. Since the price and its demands of ASFs in the community are high, thus every product of livestock is intended for market purposes. As the result, the consumption of ASFs is hampered among children. Similarly, they are more interested to save money so as to purchase and construct urban houses. The participants reported that the livestock and their products are produced for the market purpose as explained by the following quote.

*The cost of the animals and ASFs have sky-rocketed due to a mismatch between supply and demand of ASFs*, *and the presence of good communication access among the different towns of the region and the country*. *Thus the farmers get used to the sale of the livestock products and eventually save money to construct houses in the nearby urban areas (KII*, *male*, *age 32 years)*.

On the other hand, participants remarked that the availability of ASFs except for milk and meat is not a key challenge at their local market places, but their costs are extremely expensive.

*We don’t have all types of ASFs at home though chicken*, *butter*, *and eggs are abundant in the local markets*. *However*, *due to their cost*, *we don’t purchase these food products and provide for our children (FGD*, *father*, *age 28 years)*.

An FGD participant added her feelings as follows:

*If you have money*, *you can get an animal and ASFs in our local market*. *Livestock and livestock products such as egg*, *chicken*, *butter*, *sheep*, *and goats are found here except meat and milk*. *But*, *their price is too expensive to afford by the poor (FGD*, *mother*, *age 25 years)*.

#### Mothers’ workload to herd livestock

Although livestock owners have the opportunity to provide animal products to their children, herding livestock was a big chore for mothers in the study area. In the study communities, livestock production is an extensive system and the forage and watering site is far from the home of the household. Thus, livestock herding is a big task for mothers because it increases the maternal labour demand that causes inadequate child care, reduces child dietary diversity and ASFs consumption. IDI participant indicated that:

*There is no difference between high and low livestock ownership with regard to meat consumption. Those who own 70–80 sheep and goats never slaughtered for their children. Rather mothers are busy in keeping their livestock and have no time to consume foods on time for themselves and their children (IDI, mother, age 35 years)*.

FGD participants reported that all activities concerning livestock business mainly run by mothers and female adolescents as stated in the following quote:

*Mothers decreased their food consumption for themselves and to their children because of the high work burden in keeping, feeding and watering their livestock. In our community, most of the livestock management is the task of adolescents and mothers (FGD, mother, age 27 years)*.

#### Household income

The respondents revealed that the presence of low and scarce food and financial resources has affected children’s capacity to consume ASFs. ASF consumption in children was linked to household income status. Income generated from the sales of livestock and their products was the main source of money to cover school, food, and other expenses as described in the following quote.

*We sell the livestock and their products so as to cover the expenses for foods*, *schools*, *agriculture inputs and health care (IDI*, *mother*, *age 31 years)*.

The participants described that the prices of ASFs are high and they get benefit from the sales of ASFs to cover other home expenses by purchasing more and lower price food products.

*Livestock and their products are produced for market purposes*. *The money earned from selling one liter of butter is sufficient enough to purchase threefold of palm oil (IDI*, *mother*, *age 28 years)*.

The animal and ASFs are produced as compensation to crop failure during the drought seasons. The community purchases the food and other home expenses from the sales of livestock and their products.

*The livelihood of the community is dependent on crop and livestock production*. *Since there is a recurrent drought in the community*, *animals and their products are the insurances for the crop failure*. *Thus*, *sales from animals and animal products are used to cover food and other household expenses at times of crop failure (KII*, *female*, *age 25 years)*.

#### Low milk production

Study participants reported that there is no accessibility of milk and milk products at their most convenient area such as local marketplaces. The availability of underperforming livestock breeds and lack of animal feed and water has resulted in lesser ASFs production and hence decreased children’s milk consumption as described by the following quote.

*This area is the most drought-prone area*. *Animal feed and water are the main problems of this community*. *Farmers do have more than two or three dairy cows of poor breeds that could give birth at an interval of four years*. *Thus*, *we couldn’t get milk at any time at home or in the nearby local market*. *We only benefited from the relative drought resistant sheep and goats*. *The scarcity of grazing land due to their conversion into crop farms is also another challenge for improved animal and ASFs production (IDI*, *male*, *age 37 years)*.

#### The poor linkage between the health and agriculture sectors

Although health experts promote child dietary diversity through the consumption of ASFs, the agriculture sector mainly focused on increasing the farmers’ income by selling the agricultural products including ASFs. It was reported that the low consumption of ASFs is fueled by agricultural experts, in that they only encourage the farmers to produce a surplus and sell livestock and their products to save money in banks.

*Training provided by the agricultural sector mainly focused on the increase of production and productivity rather than on consumption*. *They encourage us to produce high quality and quantity livestock products so as to obtain money from the selling of animals and animal products*. *For instance*, *farmers have introduced the best performing local breed cows (known as Begait)*. *However*, *the agriculture sectors always insist on farmers to sell the milk/butter and save money in their bank account (KII*, *male*, *age 52 years)*.

#### Social norms and beliefs

Participants stated that children may not get access to ASFs during the fasting season although households own milking cows and goats due to fear of contamination of cooking utensils and for fear of violation of religious fasting rules and procedures.

*…during the major fasting periods of the Ethiopian Orthodox Church*, *no one above seven years of age consume ASFs except women who are lactating in the first few weeks of delivery*. *Thus*, *no animals are slaughtered during the fasting periods of the Ethiopian Orthodox Church*. *Though children could consume during the fasting days*, *households do not encourage the availability of ASFs for fear of contamination of cooking utensils (FGD*, *male*, *age 26 years)*.

The participants also indicated that avoiding meat and honey for children below two years of age is common in their localities. They reported that children have no teeth to chew meat and honey has a negative influence on their speaking competence as it is stated in the following quote:

*We don’t give meat and honey to our children of two or less than two years old*. *Children of this age don’t grow teeth to chew meat*, *and honey has an effect on their speaking capability*. *Honey is believed to cause lisping (FGD*, *lactating mother*, *age 33 years)*.

There was also a traditional perception that certain ASFs should be served first to the father before anybody in the family.

*…no child and/or mother is allowed to consume chicken in the absence of the husband because traditionally it is believed that chicken is the most respected food which should be served to the father first (IDI*, *lactating mother*, *age 34 years)*.

Opposing ideas were noted concerning the culture of chicken for husband tradition. Some study participants reported that currently the practice is fading away and any food in the households including chicken is provided equally without any discrimination among the whole families. An IDI participant indicated that:

*Traditionally*, *chicken is mainly prepared for the husband*. *However*, *currently*, *the tradition is not commonly practiced in the communities (IDI*, *male*, *age 32 years)*.

### Perceived facilitators to consumption of ASFs

#### Availability of nutrition expert

The nutrition experts are helping the communities through promoting better diet and nutrition to reduce malnutrition mainly among mothers and children. They provide training on the area of nutrition sensitive interventions such as home gardening; poultry production, water, sanitation and hygiene services; utilization of insecticide treated bed nets etc. They also participate in nutrition specific interventions specifically the essential nutrition actions such as infant and young child feeding, maternal nutrition and micronutrient deficiencies.

Participants reported that awareness creation is provided to mothers on feeding practices to children from the study communities. The presence of a nutrition expert at the district level has facilitated the provision of nutrition education sessions at the community level through the health center and health post staff.

*We have been given training about maternal and child nutrition by the nutrition experts of our district and others*. *Though the education was not specific to ASFs*, *we are aware of the importance of food diversity to mothers and children*. *Thus*, *we advise women who are lactating to feed their children such as porridge*, *soup*, *milk*, *egg*, *fruits and vegetables (KII, woman, age 28 years)*.

#### Cooking demonstrations

Regardless of mothers’ practice at home, health extension workers organize cooking demonstrations to train mothers on the preparation of complementary foods and feeding practices of children as explained by the following quote.

*We received training about the complementary feeding of children when the children reach six months old*. *During the cooking demonstrations*, *we are educated on the preparation of porridge with three portions of cereals and one portion of legumes (3*:*1 ratio) and adding vegetables and milk (FGD*, *lactating mother*, *age 26 years)*.

#### Government efforts to reduce child stunting

Participants identified the government led multi-sector intervention program designed to improve child feeding and reduce stunting, the Seqota Declaration to end child undernutrition in Ethiopia by 2030 and the presence of development partners such as Relief Society of Tigray (REST) as the facilitators of the consumption of ASFs.

*The Sustainable Undernutrition Reduction in Ethiopia (SURE)*, *the Soqota Declaration and REST are working in improving child breastfeeding*, *complementary feeding*, *and dietary diversity and others*. *They encourage mothers to grow home gardens*, *provide improved seeds or poultry to the poor*, *support water management and food security programs (KII*, *male*, *age 36 years)*.

#### Livestock in-kind credit programs

Participants reported that animals such as poultry and dairy cows are commonly distributed to poor farmers of the community with five years credit to empower poor farmers by increasing their wealth and food production level often materialized by increasing agricultural productivity and off-farm income. The in-kind farm credit program provides households with the opportunity to access ASFs and feed their children.

*We have been provided with improved dairy cows and poultry by credit from the government*. *I have taken five improved chicken and hence have access to eggs for my children*. *My sister has been given begait breed (improved dairy cow) and she gets milk for her child and butter for the market (FGD*, *mother*, *age 35 years)*.

#### Livestock ownership

During the discussion, participants said that farmers who raised particularly poultry and improved dairy cows have the opportunity to increase their egg and milk consumption and to improve their child nutrition. One female participant noted:

*…during the time of training*, *it is easy to promote and insist mothers utilize their agricultural products for their children*. *If mothers own poultry*, *we tell them to give egg and if they have a dairy cow*, *the child has a better chance to get cow milk (KII*, *female*, *age 30 years)*.

Study participants mentioned that there is no habit of selling fresh milk in the rural communities. They only sell butter after extracting from the fermented milk. Weak or physically injured animal is also used for their family consumption. An FGD male participant stated that:

*If we have a dairy cow*, *we don’t have the habit of selling milk rather we use it for home consumption after separating the butter*. *If there is also weak and/or physically injured animal*, *it is slaughtered for home consumption (FGD*, *father*, *age 42 years)*.

## Discussion

This qualitative study explored the barriers, and facilitators for ASF consumption among 6–23 months old children in rural communities of Northern Ethiopia. The findings indicated that ASFs consumption among infants and young children were very low in the study communities. Barriers to ASF consumption included consideration of the livestock products as sources of cash/income, poor nutrition knowledge, cost of ASF, mothers’ workload to herd livestock, low household income, low milk production, the poor linkage between the health and agriculture sectors and social norms and beliefs. On the other side, the presence of nutrition experts at all levels, use of cooking demonstrations, the presence of government programs to reducing child stunting, provision of livestock in-kind credit programs, and household livestock ownership were the perceived facilitators of ASF intake in the study context.

Low intake of ASF among 6–23 months old children was stated in the study communities. A similar finding was also found in other areas of Ethiopia where foods of animal origin like meat, egg, and dairy products were a rare component in the children’s diet [28]. Complementary foods of children in Ethiopia mainly are based on cereals and legumes and mostly an extension of family foods which are made from staple cereals or starchy tubers such as maize, sorghum, millet, oat, teff, potato, and barley and which are served as gruel, porridge and other forms of home-made breads locally known as *kitta*, *and dabo* [13].

According to the findings of Ethiopia National Food Consumption Survey [29], it was reported that the consumption of any flesh foods was low in children across the different regions, accounting for less than 1% of total weight consumed. This is also consistent with the current study findings where ASFs were destined for market and household income than their home consumption by the family members. A qualitative result in Ghana [30] also confirmed that livestock and their products were used mainly as a source of bank saving rather than for consumption and nutrition. Similarly, a study in Malawi reported that cash was ranked as the most important reason for keeping cattle in smallholder farmers [31].

Low nutritional knowledge was found to be a barrier for children’s ASF consumption in the study area. A similar finding was also reported in Ghana, which demonstrated that caregivers’ nutrition knowledge and feeding attitudes were significantly associated with both the household diet and the intake of ASFs by 2–5 years old children [32]. Nyantakyi-Frimpong *et al*., [30] Colecraft *et al*., [33] and Mukta *et al*., [34] also added that low ASFs consumption was due to the lack of information on the nutritional benefits of these foods. Thus, knowledge about the importance of ASF should be addressed through appropriate nutrition education [35]. A study of Ethiopian mothers found that they were unwilling to feed pre-school children with meat or other animal source foods because they believed that children cannot digest these food items [36]. Abebe *et al*. [37] documented considerable maternal knowledge gaps about complementary feeding practices, especially regarding meal frequency and dietary diversity in northwestern Ethiopia. Caregivers, who lack the understanding of the importance of providing children with certain foods such as ASFs, may not provide these to their children even when the food is available in the household. Behavioral Change Communication (BCC) interventions that seek to improve caregivers’ nutrition knowledge can be effective at improving feeding practices of infant and young children.

Another barrier of the ASFs consumption among children was the cost of ASFs. Although participants revealed that there is access to animal and ASFs particularly egg, chicken, butter, sheep and goats in their local market, the costs of these ASFs are often prohibitively priced which doesn’t encourage purchasing by the poor households to afford the price. Previous works have also reported reduced ASFs consumption in children for cost-related reasons [38–42]. There is suggestive evidence that consumption of ASFs was associated with improving child nutritional outcomes [41, 43, 44]. Therefore, increasing consumption of ASFs in sufficient quality and quantity and at low prices is desirable to improve child nutritional status.

Comparable to the current study, household income has also been consistently reported in many other contexts as barriers for ASFs consumption [30, 33, 40, 45–49]. Support for the promotion of income generation activities for caregiver households and the establishment of financial service facilities for loans for entrepreneurial development was reported as the possible suggested intervention to these constraints [33].

Similarly, mothers’ workload to herd livestock is another barrier which increases physical labor and demands much time to livestock management. As the result, women have no time to prepare foods and care and feed their children. The present study is consistent with a study conducted by Njuki *et al*., [50] who reported that maternal workload associated with livestock production could impact adversely on caregiver’s income and on caregiver’s time and workload, with detrimental effects on their caregiving activities and child nutrition in the household. It was also reported from a review by Randolph *et al*., [51] where labor allocated to livestock can increase total household labor demands, mainly for women, and reduce the quality of care and feeding of young children, negatively influencing their nutritional status.

The present study demonstrated that the community’s beliefs and norms were barriers to ASF consumption among children from the study communities. Avoidance of ASFs during fasting seasons and prioritizing best foods for fathers was reported by most of the study participants. The fasting period of the Ethiopian Orthodox Church accounts for more than 150 days per year and no ASF is consumed by most adults practicing Orthodox Christianity [28, 47, 48, 52]. During this period, ASF may not be prepared at home and be available for children because the mother/caregivers are not willing to prepare non-fasting foods for their children during the fasting season due to their fear that it could contaminate utensils used for cooking family foods [28, 46, 53, 54]. This result is also supported by a quantitative research result by Kim *et al*. [46] who indicated that during the Lent fasting period, only one quarter of the children consumed ASF and one third of households had any ASF available in the house, although 80% of the study households owned livestock animals (chickens, cows, goats, and/or sheep). Furthermore, the present study witnessed that priorities were given for fathers for certain ASFs such as chicken and meat, and avoidance for children below two years was reported although not all participants agreed on this view point. Similar research findings were reported in Ghana by Nyantakyi-Frimpong *et al*. [30] and Colecraft *et al*. [33] who stated that some ASFs are more favored to fathers and old people compared to the other families in the community. The perception of our study participants about the avoidance of meat to children is also supported by Colecraft *et al*. [33] and Nakachew *et al*. [55]. These studies reported that meat was too tough for children to chew suggesting the need for behavior and attitude change among the study communities.

Low production and productivity of milk was also reported as a barrier to the consumption of animal products in the study areas. The low milk production is believed due to low feed access and poor livestock productivity. Poor productive and reproductive potential of Ethiopian indigenous cows was reported by Kumar *et al*. [56] and Mulugeta and Belayeneh [57], and the seasonality in agricultural production and in the supply of agricultural commodities, including ASF, is due to climatic and most importantly, rainfall patterns which is linked with the production of animal feed [48, 58]. As a result, the milk and milk products are not available and accessible to the children since farmers of the study communities rely heavily on rain-fed agriculture and thus face persistent water problems during the hot dry season. The major constraints that limit cattle production and productivity in Ethiopia are genetic resources and shortage of feed and water [59, 42].

The present study also confirmed that experts from the agriculture sector encourage the community to have market-oriented production rather than child consumption. This finding is comparable with a previous study in Africa [60] which indicated that specialists from the agriculture sector may focus on technical challenges of production and productivity and may have poor knowledge outside of their fields of expertise. Findings by Girmay *et al*. [61] added that at district level, there was no effective linkage between the health and agriculture sectors and each sector focused only on its own priorities rather than working in collaboration to achieve local nutrition objectives.

The presence of nutrition experts at each district and the health and agriculture extension workers at the local community level facilitated the consumption of ASFs to children due to the provision of health and nutrition services such as training and counseling to the community. Similar to our findings, Girmay *et al*. [61] showed that the presence of extension workers who were actively engaged in nutrition had facilitated the successful delivery of nutritional services to the community. A study from other regions of Ethiopia added that most mothers/caretakers get training from Health Extension Workers on the preparation of diversified food from the locally available food items [55].

The provision of training on cooking demonstrations to community members was identified as one of the facilitators for ASFs consumption. Caregivers were trained by the health extension workers about child feeding through a practical demonstration on the preparation of nutrient and energy dense complementary from locally available ingredients. Promotion of local production of complementary foods and training of health workers and health extension workers on the preparation of enriched complementary foods is one of the main initiatives of the Ethiopian National Nutrition Program [19]. Such practices are believed to improve caregivers’ knowledge of infant and young child feeding practices [62–66].

The presence of government-led child stunting reduction programs in the county was reported as a facilitator to consume ASF by children in the communities. According to the National Nutrition Program, in 2016 there was a targeted plan of reducing child stunting prevalence among under-five children from 40% to 26% by 2020 [19]. One of the initiatives to achieve this plan is through promoting and protecting optimal breastfeeding practices and building the capacity of service providers on complementary feeding for children aged 6–23 months [19]. Moreover, the presence of nutrition promotion program partners such as sustainable undernutrition reduction in Ethiopia (SURE) and the Soqota Declaration programs were raised as main facilitators to the consumption of ASFs by children in the communities. In line with this finding, community networks and the increasing engagement of development partners or non-governmental organizations (NGOs) in nutrition were reported as strong facilitators for good health and agriculture linkages at community level [61]; and the nutrition-specific changes, represented in most cases by solidly implemented government or NGO supported interventions at the community level [67].

The presence of livestock in-kind credit program was reported to be opportunities to facilitate the consumption of ASFs among children in the study area. In line with this finding, livestock in-kind credit was stated as a possible intervention for the barriers that decline the consumption of ASFs among women of reproductive age in Ghana [30]. The finding aligns with a study done in Rwanda [68] and Ethiopia [69] which has shown that these interventions truly increase ASF consumption and improve nutritional status.

Livestock ownership was reported as an opportunity to consume ASFs by children in the study area. Similarly, livestock ownership was reported as a facilitator to the consumption of ASFs among communities of other studies [41, 70]. On the contrary, livestock ownership was not significantly associated with children’s odds of ASF consumption, child height-for-age z-score or odds of stunting in the Luangwa Valley [71]. This difference could occur due to the total livestock holdings in the population and market-based production of livestock. Livestock ownership coupled with nutrition education or behavior change communication interventions seemed to ensure consumption of ASFs for nutritional benefits [46].

### Strengths and limitations

The inclusion of various study groups to find out the barriers and facilitators for optimal ASF consumption was the major strength of this study. Trustworthiness was maintained through open dialogue and discussion with researchers. Although, it is understood that there is considerable intra- and inter-region variability, transferability to other communities in the country was maintained through the selection of the study participants from two districts and six Tabias representing diverse social environments and the Seqota Declaration interventions areas. Though the KII, IDI, and FGDs were carefully carried out, it is difficult to know to what extent the participants over-reported or under-reported their positive or negative behaviors. The possibility of social desirability bias as parents (mothers and fathers) would want to be perceived as “good actors” can’t be ruled out. However, it was tried to minimize this potential bias by reminding participants that discussions were anonymous as it was described in the methods section; data were gathered from a variety of participants and comparing responses. Some key details might have been also lost when the interviews were translated it into English. But to minimize such impacts, the audio-recorded materials were transcribed each interview and discussion word-for-word in the local language, Tigrigna, and then it was translated into English. The translations were verified by listening to the recordings while re-reading the transcripts.

### Conclusions

Low consumption of ASF among 6–23 months old children was reported from the study of rural communities of Northern Ethiopia. Barriers of ASF consumption included the lack of nutrition knowledge, cost of ASF, mothers’ workload to herd livestock, low household income, low milk production, the poor linkage between the health and agriculture sectors and social norms and beliefs. The facilitators to consume ASFs were the presence of nutrition experts, cooking demonstrations, the presence of government-led child stunting reduction programs, the presence of livestock in-kind credit program, and livestock ownership. Therefore, activities which will improve the consumption of ASFs among 6–23 months old children including social and behavioral change communication to raise awareness about the health and nutritional benefits of ASFs consumption, strengthening the off-farm income generation activities, scaling up of the cooking demonstrations and strengthening the linkage between the health and agriculture sectors are recommended to promote ASF consumption among children 6–23 months old children in rural communities of Northern Ethiopia are recommended.

## Supporting information

S1 Table Guiding questions for focus group discussions and qualitative interviews in English. (PDF)

S2 Table Guiding questions posed to participants during the focus group discussions and interviews in local language, Tigrigna. (PDF)

## Abbreviations

ASF: animal source foods
FGD: focus group discussions
IDI: in-depth interviews
NGO: non-government organization
