# Correction: Why are animal source foods rarely consumed by 6-23 months old children in rural communities of Northern Ethiopia? A qualitative study

**DOI:** 10.1371/journal.pone.0230527

**Published:** 2020-03-11

**Authors:** Mekonnen Haileselassie, Getachew Redae, Gebretsadik Berhe, Carol J. Henry, Michael T. Nickerson, Bob Tyler, Afework Mulugeta

The images for Figs 2 and 3 are incorrectly switched. The image that appears as Fig 2 should be Fig 3, and the image that appears as Fig 3 should be Fig 2. The figure captions appear in the correct order.

**Fig 2:**
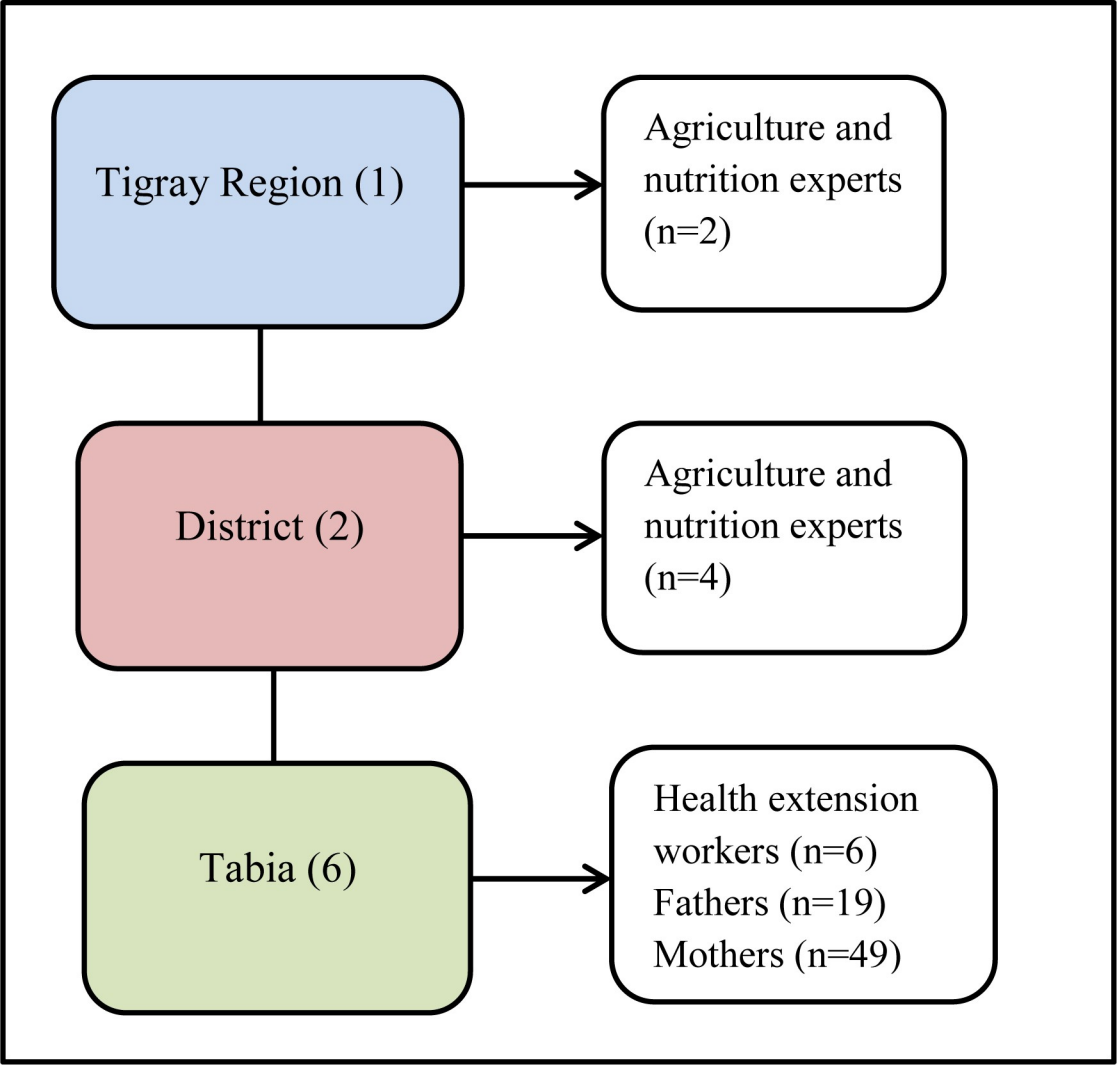
Schematic representation of the sampling procedure for the qualitative study in Tigray, Northern Ethiopia, 2019.

**Fig 3:**
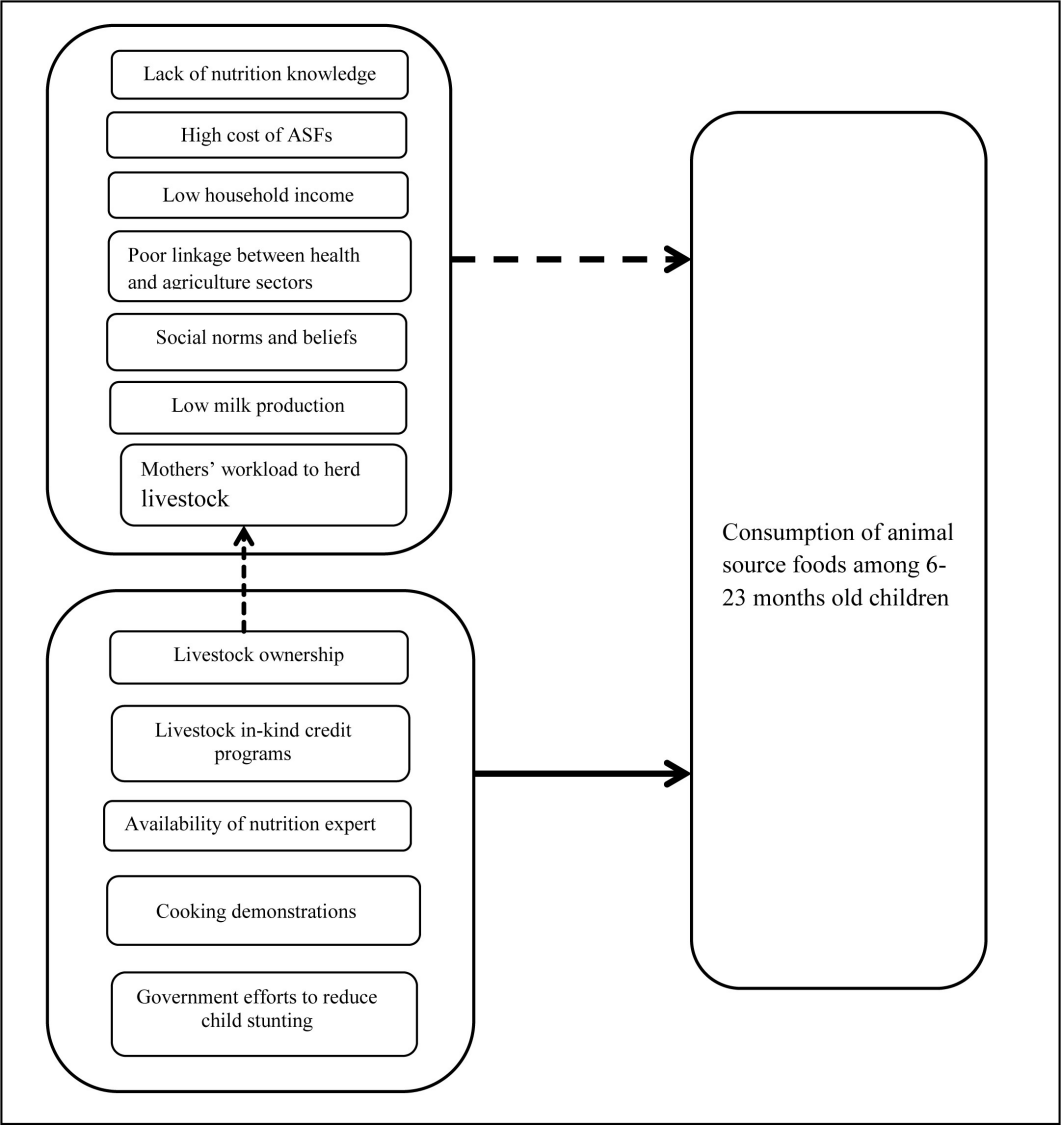
Conceptual framework describing the barriers and facilitators to the consumption of animal source foods among 6–23 months old children in rural communities of Northern Ethiopia. Note: the solid line indicates positive influence and dashed lines show the negative influence on the consumption of ASFs among 6–23 months old children. Livestock ownership has positive effects for the consumption of ASFs among children and it has also negative impact on mothers’ workload to herd livestock.
