# Supplementary material for: Why are animal source foods rarely consumed by 6-23 months old children in rural communities of Northern Ethiopia? A qualitative study
Source: PLoS One. 2020 Jan 8;15(1):e0225707. doi: 10.1371/journal.pone.0225707 (PMC6948827; doi:10.1371/journal.pone.0225707)
Supplement: S1 Table — (PDF) [file pone.0225707.s001.pdf]

S/N	Questions
1	What are the common livelihoods of the people here?
2	What are the major foods produced in this area, and for what purpose?
3	What are the common nutrition problems in the community among 6-23 months old children?
4	Can we now discuss the complementary foods that are usually considered healthy nutritious foods given to infants and young children in this community?
5	How do you evaluate the consumption level of animal source foods (ASFs) among 6-23 months old children in this community?
6	In your opinion, how should 6-23 months old children is fed ASFs?
7	Which ASFs do you consider the most nutritious food for your child? Why?
8	How do you get ASFs for your children of 6-23 months old?
9	What are the barriers to access and utilization of ASFs among 6-23 months old children?
10	What could be the possible facilitators in delivering ASFs to children in this community?
11	What are the successful strategies that promote ASFs consumption to children in this community?
12	Which animal types provide more ASFs to the household?
13	What are the main incomes of livestock in this community?
14	Do you purchase or sell animal and ASFs from/to market? If yes which ASFs and if no why?
15	How do you evaluate the price and availability of animal and ASFs in the market?
16	Which animal and ASFs are high/less price in the market? Why?

17	What is the collaboration status between agriculture and health sectors in promoting ASFs consumption in children?
18	How do you learn about ASFs feeding to children?
