# Supplementary material for: Why are animal source foods rarely consumed by 6-23 months old children in rural communities of Northern Ethiopia? A qualitative study
Source: PLoS One. 2020 Jan 8;15(1):e0225707. doi: 10.1371/journal.pone.0225707 (PMC6948827; doi:10.1371/journal.pone.0225707)
Supplement: S2 Table — (PDF) [file pone.0225707.s002.pdf]

ተ.ቁ.	ሕቶታት
1	መነባብሮ ሕ/ሰብ ኣብ ምንታይ ዝተመስረተ እዩ?
2	ኣብ ከባቢኩም ዝምረቱ ዋና ዋና ዘራእቲ/ምግብታት ብዓይነቶምን ረብሓኦምን ታይ ይመስሉ? (ንውሽጢ ዓዲ ዕዳጋ፤ ንምግቢ፤ ንወፃኢ ዕዳጋ ንካልኦትን)
3	ኩነታት ስነ-መዓዛ ኣብ ቆልዑን እነታትን ኣብ ከባቢኩም እንታይ ይመስል?
4	ናይ ስነ መዓዛ ትሕዝቶኦም ዝሐሹ እዮም ብምባል ንሕፃናትን ቆልዑን ዝዋሃቡ ዓይነት ምግብታት እንታይን እንታይን እዮም?
5	ኣብዙ ከባቢ ሕፃናት (6-23 ወርሒ) ናይ እንስሳት ተዋፅኦ ኣመጋግበኦም እንታይ ይመስል? (ከመይ ትርእዎ)?
6	ናይ ናይ እንስሳት ተዋፅኦ ኣብ ምምሕያሽ ስን-መዓዛ ቆልዑ ረብሓ ኣለዎ ኢልኩም ዶ ትኣምኑ? ብከመይ ቆልዕት ናይ እንስሳት ተዋፅኦ ይምገቡ?
7	ንሕፃናት ኣየናይ ናይ ናይ እንስሳት ተዋፅኦ እዩ እቲ ዝሓሸ ስነ-መዓዛ ትሕዝትኡ? ንምንታይ?
8	ናይ እንስሳት ተዋፅኦ ንሕፃናት ደቁኩም ካበይ ትረኽቡ?
9	ናይ እንስሳት ተዋፅኦ ምግብታት ንምርካብ ይኩን ንምጥቃም ዘለው ማሕልካታት እንታይ እንታይ እዮም?
10	ናይ እንስሳት ተዋፅኦ ምግብታት ንምርካበ ይኩን ንምጥቃም ዘለው ዕዱላት ይኹን ፃዕርታት እንታይ ይመስል?
11	ኣብ ሞንጎ ሕ/ሰብኩም፤ እቶም ዕውታት ዝባሃሉ ንእንስሳት ተዋፅኦ ንምጥቃም ዝሕግዙ ስራሕቲ እንታይ እንታይ እዮም?
12	ኣይነኦም ዓይነት እንስሳት ዝሓሸ ናይ እንስሳት ተዋፅኦ ንስድራቤት የበርክቱ?
13	ነዚ ሕ/ሰብ፤ ካብ እንስሳት ዝርከብ ዋና ዋና ረብሓን ኣታዊን እንታይ ይመስል?
14	ናይ እንስሳት ተዋፅኦ ካብ ዕዳጋ ትገዝኡ ወይ ትሸጡ ዶ? እወ እንተኾይኑ መልስኹም እንታይ እንታይ ትሸጡን ትዕድጉን? ኣይፋሉን እንተኾይኑ ልምንታይ?

15	ናይ እንስሳት ተዋፅኦ ምግብታት ንምርካበ ይኩን ንምጥቃም ዘለው ዕዱላትን ዋገኦምን እንታይ ይመስል?
16	ኣይንኡ ናይ እንስሳት ተዋፅኦ ኣብ ዕዳጋ ይሓስር ወይ ይኸብር? ልምንታይ?
17	ናይ ሕፃናት ስነ-ማዓዛ ንምምሕያሽ ምስ መዳርግቲ ኣካላት ብፍላይ ኣብ ሞንጎ ሕርሻን ጥዕናን ዝስርሑ ስራሕቲ ኣብ ዙርያ ናይ እንስሳት ተዋፅኦ ምጥቃም ኣብ ህፃውንቲ ቆልዑ እንታይ ይመስል?
18	ብዝዓባ እንስሳት ተዋፅኦ ንሕፃናት ምጥቃም ብኸመይ ንኣዶታት ይኹን ንሕ/ሰብ ይምሓላለፍ?
